# Aspects related to health literacy, self-care and compliance with treatment of people living with HIV^*^

**DOI:** 10.1590/1980-220X-REEUSP-2022-0120en

**Published:** 2022-10-17

**Authors:** Mônica Alice Santos da Silva, Morgana Cristina Leôncio de Lima, Cynthia Angélica Ramos Oliveira Dourado, Maria Sandra Andrade

**Affiliations:** 1 Universidade de Pernambuco, Faculdade de Enfermagem Nossa Senhora das Graças, Programa Associado de Pós-Graduação em Enfermagem. Recife, PE, Brazil.; 2 Universidade Estadual de Paraíba. Campina Grande, PB, Brazil.

**Keywords:** Health Literacy, Medication Adherence, Self Care, Nursing, HIV, Alfabetización en Salud, Cumplimiento de la Medicacíon, Autocuidado, Enfermería;, VIH, Letramento em Saúde, Adesão à medicação, Autocuidado, Enfermagem, HIV

## Abstract

**Objective:**

to verify the relationship between health literacy, compliance with antiretroviral therapy and self-care of people living with HIV.

**Method:**

this is a cross-sectional study, developed between January and July 2019, using validated scales on health literacy (SAHLPA), compliance (CEAT-HIV) and self-care (EACAC).

**Results:**

a total of 303 people enrolled in three HIV outpatient care services participated in the study, with a satisfactory level of literacy (52.5%), excellent level of self-care (62.9%) and strict compliance with antiretroviral therapy (57.1%). The illiterate had insufficient medication compliance, when compared with the literate (PR = 1.17). Strict compliance was significant for self-care (p-value < 0.001). A higher risk ratio for illiteracy was associated with females, people with elementary education, who receive benefits, with an income of up to one minimum wage, not having the habit of seeking health information and longer use of ART.

**Conclusion:**

a relationship was identified between literacy and insufficient compliance. The risk for insufficient medication compliance increases as self-care declines. Social measures that reduce inequities can contribute to improving care for people living with HIV.

## RESUMO

**Objetivo:**

verificar a relação entre letramento em saúde, adesão à terapia antirretroviral e autocuidado de pessoas vivendo com HIV.

**Método:**

trata-se de estudo transversal, desenvolvido entre janeiro e julho de 2019, por meio de escalas validadas sobre letramento em saúde (SAHLPA), adesão (CEAT-HIV) e autocuidado (EACAC).

**Resultados:**

participaram do estudo 303 pessoas cadastradas em três serviços de atendimento ambulatorial-HIV, apresentando grau satisfatório de letramento (52,5%), ótimo nível de autocuidado (62,9%) e adesão estrita à terapia antirretroviral (57,1%). Os não letrados apresentaram adesão medicamentosa insuficiente, quando comparados com os letrados (RP = 1,17). A adesão estrita foi significativa para o autocuidado (p-valor < 0,001). Uma maior relação de risco para o não letramento esteve associada ao sexo feminino, pessoas com ensino fundamental, que recebem benefícios, com renda de até um salário mínimo, não ter hábito de buscar informações em saúde e maior tempo de uso da TARV.

**Conclusão:**

identificou-se relação entre o letramento e a adesão insuficiente. O risco para adesão medicamentosa insuficiente aumenta à medida que o autocuidado diminui. Medidas sociais redutoras de iniquidades podem contribuir para melhoria da assistência a pessoas que vivem com HIV.

## RESUMEN

**Objetivo:**

verificar la relación entre la alfabetización en salud, la adherencia a la terapia antirretroviral y el autocuidado de personas que viven con VIH.

**Método:**

se trata de un estudio transversal, realizado entre enero y julio de 2019, utilizando escalas validadas de alfabetización en salud (SAHLPA), adherencia (CEAT-HIV) y autocuidado (EACAC).

**Resultados:**

Participaron del estudio 303 personas inscritas en tres servicios de atención ambulatoria de VIH, con nivel de alfabetización satisfactorio (52,5%), nivel de autocuidado excelente (62,9%) y adherencia estricta a la terapia antirretroviral (57,1%). Los analfabetos tenían adherencia insuficiente a la medicación, en comparación con los alfabetizados (RP = 1,17). La adherencia estricta fue significativa para el autocuidado (valor p < 0,001). Una mayor razón de riesgo para el analfabetismo se asoció con el sexo femenino, personas con educación básica, que perciben beneficios, con ingresos de hasta un salario mínimo, que no tienen el hábito de buscar información de salud y mayor uso de la TARV.

**Conclusión:**

se identificó una relación entre la alfabetización y la adherencia insuficiente. El riesgo de mala adherencia a la medicación aumenta a medida que disminuye el autocuidado. Las medidas sociales que reducen las desigualdades pueden contribuir a mejorar la atención de las personas que viven con el VIH.

## INTRODUCTION

Despite the availability of effective prevention measures and free treatments, the goal of controlling HIV infection as a public health problem is still considered a global challenge. In Brazil, although there has been a decrease in the detection rate since 2012, from 22.0/100 thousand inhabitants to 14.1/100 thousand in 2020, representing a decrease of 35.7%, added to the decrease in 29.9% in the mortality coefficient, standardized in the year 2020, 32,701, new cases of infection were reported. Of the new cases of infection, 25% are concentrated in the Northeast region^(1)^.

The drugs available for HIV treatment have undergone changes over time, allowing for an extension of life with quality. The pathology is no longer a condition incompatible with life and is now considered a chronic disease. However, In order for treatment to reach its intended purpose, the person living with HIV (PLHIV) needs to understand the concepts related to living with the infection, strictly comply with the prescribed therapy and develop self-care that provides a link in the cascade of continuous care^(2)^.

Considering the importance of inserting PLHIV at the center of care, global organizations highlight the literacy construct as essential for public health, as it is related to people’s ability to access, process and make use of health information. Its proper development transcends individual conditions, resulting from an interaction between health services, professionals in these environments and users. It is also considered an important reducer of inequities, since accessing information and health services is a right for all, guaranteed by law, but not always achieved. Knowing the impacts of health literacy (HL) in this population is essential to propose effective interventions^(3,4)^.

Systematic review studies emphasize the importance of developing research that elucidates the relationships between HL and health outcomes related to living with HIV, such as viral load remission and fewer missed appointments^(5)^. Health interventions with PLHIV that use HL as theoretical support have shown improvement in treatment compliance^(6)^; however, more research studies on the relationships between HL and health outcomes are needed^(7)^. Although these studies refer to the reality of other countries, the situation in Brazil may be even more deficient, since HL is not part of public policies related to HIV or other chronic pathologies, and there is no population study that allows the comparison of data between different regions of Brazil or the results of the effectiveness of interventions^(8)^.

In the case of PLHIV, there is a historical construction related to the disease, in which infected people suffer from the impacts of health determinants and conditions and stigmas on the pathology that lead to less access to services and a smaller social support network^(9)^. In this sense, identifying low HL provides health professionals with individualized consultations and health education actions aimed at understanding difficulties that may prevent the adoption of self-care and compliance with drug therapy. In this context, the following research question was elaborated: what is the relationship between HL, self-care and compliance with antiretroviral drugs in PLHIV?

Checking the relationship between HL, self-care and compliance with the treatment of PLHIV is of paramount importance, since the achievement of longevity and quality of life proposed by antiretroviral therapy can only be achieved through an active self-care attitude of those who make use of therapeutic resources^(2)^. In the same sense, disease control is the central proposal of health policy aimed at HIV, depending on the results achieved by people undergoing treatment. In turn, viral remission is only guaranteed by the continuous use of drugs and is linked to the motivation to comply with the proposed therapy.

Given the above, the study aimed to verify the relationship between HL, compliance with antiretroviral therapy and self-care of PLHIV.

## METHOD

### Study Design

This is a cross-sectional study. We used the recommendations from STrengthening the Reporting of OBservational studies in Epidemiology (STROBE).

### Local

The study was carried out in three HIV Specialized Care Services (SCS) in the city of Recife, Pernambuco, Brazil. To preserve identification, the services were classified into SCS 1, 2 and 3. These units, a reference in HIV care, together provide assistance to a population of 11,082 people, and are the ones that concentrate the largest number of care in the state of Pernambuco.

### Population

The population consisted of PLHIV enrolled in SCS in the aforementioned study municipality.

### Selection Criteria, Sample Definition and Study Variables

Probability sample considered a finite population and categorical dependent variable, composed of people registered in the surveyed SCS. The prevalence of 25% for inadequate literacy was used as a reference^(10)^. For calculation, a confidence level of 95% (Zα = 1.96) was considered, p as the proportion of favorable results (0.25 or 25%), q as the proportion of unfavorable results (q = 1-p), N equal to 11,082 people registered in the SCS (finite population) and standard error of ± 5%. Stratification was carried out into three sample groups, which, in turn, were proportionally separated according to the population number found in each health unit: SCS 1, with 6,806 records, SCS 2, with 2,400 and SCS, 3 with 1,876 registered cases.

To control possible sample losses, a value of 10% was added to the initial sample. The sample was stratified into 190 interviews in SCS 1, 67 in SCS 2 and 52 in SCS 3. The losses were of 3 respondents, for not continuing the test after being called for consultation and for not having the results of exams in the system. Thus, the sample consisted of 303 people.

We included people aged 18 years and over and with more than 6 months of antiretroviral therapy (ART), visual acuity testing and adequate cognitive screening. We excluded people who self-declared not having at least 1 year of formal education or not knowing how to read, with hearing impairment that made it impossible to communicate verbally and/or receive information to perform the tests, presence of psychiatric illness without treatment or control, in addition to a compromised health condition (bedridden patients brought by ambulances, tuberculosis patients without treatment and/or debilitating clinical condition).

The predictor variables were associated with social, sociodemographic (sex, age, education level, marital status, ethnicity, occupation and income), clinical (time on ART, laboratory values of viral load and CD4T+ and use of controlled drugs) and behavioral (habit of seeking health information) characteristics. The outcome variables were related to the results in the health literacy test (literate and illiterate), self-care (good, very good, excellent self-care capacity) and compliance (insufficient, strict).

### Instruments Used for Information Collection

The Rosenbaum Pocket Visual Screening Test and the Cognitive Screening Test, with the application of the International HIV Dementia Scale (IHDS)^(11)^, were used to rule out the confounding variables reading difficulty due to visual or neurological deficit, respectively, which could prevent or interfere with the responses to the literacy test and meet the sample inclusion criteria. People who had visual acuity equal to or greater than 20/50 in the visual test and a score above 10 in the cognitive assessment were considered eligible and were interviewed.

For data collection, we used a form with sociodemographic and clinical data and the Health Literacy Test Short Assessment of Health Literacy for Portuguese-Speaking Adults (SAHLPA 50), which consists of a scale with 50 items that assess the ability to the individual correctly pronounces and understands common medical terms^(12)^, as well as the ART compliance test *Cuestionário Para la Evaluación de La Adhesión al Tratamiento Antiretroviral* (CEAT-HIV online version)^(13)^ and the Self-Care Capacity Assessment Scale (EACAC)^(14)^. The three scales are validated for use in Brazil, with adequate psychometric properties and authorized for use by their respective authors.

### Data Collection

Data collection took place between January and July 2019. Recruitment took place at random. People awaiting treatment at the outpatient clinic were invited to participate in the study voluntarily. First, screening forms and tests were applied, and then people considered eligible for the study responded to the tests.

### Data Treatment and Analysis

The collected data were entered into two databases in Microsoft Excel, exported to the EPI-INFO database, version 3.5.4, with subsequent exportation to Statistical Package for the Social Sciences (SPSS 18.0) for Windows®. There was validation by checking different data in double-entry typing. The test scores were respected, according to the authors’ guidance. For data analysis, a database was built using EPI INFO, version 3.5.4, after data validity. Then, the database was exported to SPSS, version 18, in which the analysis was performed. Percentage frequencies were calculated and the respective frequency distributions were constructed. To assess the association between literacy and compliance, self-care and compliance, and literacy and self-care, contingency tables were constructed, using Pearson’s chi-square test for independence. To assess which personal, clinical and habit factors influence literacy, cross tables and the chi-square test for independence were used. To calculate the prevalence ratio (PR), the prevalence of the most exposed group was divided by the prevalence of the least exposed group for all the variables presented. All conclusions were drawn considering a significance level of 5%.

### Ethical Aspects

The ethical precepts of Resolution 466/12, which deals with studies involving human beings, were respected. The volunteers’ decision to participate or not in the research was respected. The project was submitted to the Research Ethics Committee, having received the Certificate of Presentation for Ethical Consideration (*Certificado de Apresentação para Apreciação Ética*) in 2018, under Opinion 3,068,763.

## RESULTS

A total of 303 people participated in the study, and Table 1 shows the distribution of participant sociodemographic profile. The proportion comparison test was significant in all factors assessed (p-value < 0.001), indicating that the profile described is the most frequent in the group of people assessed.

**Table 1.** Distribution of the sociodemographic profile of people living with HIV – Recife, PE, Brazil, 2019.

**Factor assessed**	**n**	**%**	**p-value**
**Sex**			
Male	197	65.0	<0.001^1^
Female	106	35.0	
**Age**			
≤ 40	109	36.0	<0.001^1^
41–59	169	55.8	
≥60	25	8.2
Minimum–maximum	20–74		
Mean ± standard deviation	44.16 ± 10.97		
**Education level**
Elementary school	104	34.7	<0.001^1^
High school	140	46.7	
Higher education/graduate degree	56	18.6	
**Marital status**			
Single	157	51.8	<0.001^1^
Married	65	21.5	
Widow	27	8.9	
Separated	21	6.9	
Stable union	33	10.9	
**Ethnicity**			
White	72	23.8	<0.001^1^
Black	59	19.5	
Brown	162	53.5	
Others	10	3.3	
**Occupation**			
Employed	114	37.6	<0.001^1^
Unemployed	65	21.5	
Studying	17	5.6	
Retired	36	11.9	
Benefit	71	23.4	
**Income**			
Up to 1 minimum wage	238	79.1	<0.001^1^
≥ 2 minimum wages	63	20.9	

1p–value of the chi–square test for proportion comparison.

The use of controlled medication was reported by 51 (16.8%) respondents. Of those who use these drugs, 24 (47.0%) use anticonvulsants. The CD4 T cell count was in the range of 501-999 to 140 (46.2%). Viral load was undetectable for 236 (77.9%). The duration of ART use ranged from 6 months to 5 years for 123 (40.6%) of the total respondents. A total of 248 (81.8%) reported the habit of seeking health information.

It appears that 159 (52.5%) have a satisfactory level of literacy, 188 (62.9%) have an excellent level of self-care and 173 (57.1%) have strict compliance with ART. Considering compliance with treatment according to the level of literacy and self-care, it is observed that the independence test for compliance was not significant for literacy (p-value = 0.225). However, it is identified that the illiterate has a PR of (PR = 1.17) for strict compliance, when compared with the literate. Strict compliance was significant for self-care (p-value < 0.001). It is noticed that the group of patients with a good self-care score has 1.23 (PR = 2.23) more risk of insufficient compliance, when compared to the group with excellent self-care. For people who have a very good level of self-care, the risk is 0.5 greater of having insufficient compliance, when compared to the group with an excellent level of self-care (Table 2).

**Table 2.** Distribution of compliance to treatment of people living with HIV according to the level of literacy and self–care – Recife, PE, Brazil, 2019.

**Factor assessed**	**Compliance**		PR*	(95%) CI**	**p–value**
	**Insufficient**	**Strict**			
**Literacy**
Illiterate	67(46.5%)	77(53.5%)	1.17	0.91–1.52	0.225^1^
Literate	63(39.6%)	96(60.4%)	1.00	–	
**Self–care**					
Good	22(75.9%)	7(24.1%)	**2.23**	**1.67–2.97**	**<0.001** ^1^
Very good	42(51.2%)	40(48.8%)	1.50	1.13–2.01	
Great	64(34.0%)	124(66.0%)	1.00	–	

^1^p–value of Pearson’s chi–square test for independence. *Prevalence ratio; *Correlation Index.

In the analysis of self-care according to literacy level, there was a higher percentage of excellent self-care in the literate group, 102 (64.6%), but without statistical significance (p-value = 0.238) (Table 3).

**Table 3.** Distribution of self–care of people living with HIV according to literacy level – Recife, PE, Brazil, 2019.

**Literacy level**	**Self–care**			**p–value**
	**Good**	**Very good**	**Great**	
Illiterate	18(12.8%)	37(26.2%)	86(61.0%)	0.238^1^
Literate	11(6.9%)	45(28.5%)	102(64.6%)	

^1^p–value of Pearson’s chi-square test for independence.

In the distribution of the literacy level according to the sociodemographic profile, there was a greater risk ratio for illiteracy in females (p-value = 0.010), with elementary education (p-value < 0.001), who receive benefits (p-value < 0.001) and with an income of up to one minimum wage (p-value < 0.001) (Table 4).

**Table 4.** Distribution of literacy among people living with HIV in Recife according to sociodemographic variables – Recife, PE, Brazil, 2019.

**Factor assessed**	**Literacy**		PR*	(95%) CI**	**p-value**
	**Illiterate**	**Literate**			
**Age**					
≤ 40	44(40.4%)	65(59.6%)	1.00	–	0.125^1^
41–59	89(52.7%)	80(47.3%)	1.30	1.00–1.71	
≥60	11(44.0%)	14(56.0%)	1.09	0.66–1.79	
**Sex**					
Male	83(42.1%)	114(57.9%)	1.00	–	**0.010** ^1^
Female	61(57.5%)	45(42.5%)	1.37	1.08–1.72	
**Education level**					
Elementary school	86(82.7%)	18(17.3%)	15.44	5.12–46.58	**<0.001** ^1^
High school	52(37.1%)	88(62.9%)	6.96	2.26–21.29	
Higher education/graduate degree	l3(5.4%)	53(94.6%)	1.00	–	
**Marital status**					
Single	71(45.2%)	86(54.8%)	1.01	0.74–1.40	0.653^1^
Married	29(44.6%)	36(55.4%)	1.00	–	
Widow	16(59.3%)	11(40.7%)	1.33	0.88–2.01	
Separated	11(52.4%)	10(47.6%)	1.17	0.72–1.92	
Stable union	17(51.5%)	16(48.5%)	1.15	0.75–1.77	
**Ethnicity**					
White	31(43.1%)	41(56.9%)	1.00	–	0.357^1^
Black	34(57.6%)	25(42.4%)	1.34	0.95–1.89	
Brown	74(45.7%)	88(54.3%)	1.06	0.77–1.45	
Others	5(50.0%)	5(50.0%)	1.16	>0.59–2.28	
**Occupation**					
Employed	34(29.8%)	80(70.2%)	5.07	0.74–34.66	**<0.001** ^1^
Unemployed	34(52.3%)	31(47.7%)	8.89	1.31–60.39	
Studying	1(5.9%)	16(94.1%)	1.00	–	
Retired	22(61.1%)	14(38.9%)	10.39	1.52–70.81	
Benefit	53(74.6%)	18(25.4%)	12.69	1.89–85.38	
**Income**					
Up to 1 minimum wage	129(54.2%)	109(45.8%)	**2.28**	**1.44–3.59**	**<0.001** ^1^
≥ 2 minimum wages	15(23.8%)	48(76.2%)	1.00	–	

^1^p–value of Pearson’s chi–square test for independence. *Prevalence ratio; **Correlation Index.

In the distribution of degree of literacy according to clinical and behavioral variables, a higher percentage of illiteracy was identified in the group of patients using controlled medication, 27 (52.9%), and who reported interruption of ART (p-value = 0.081). Not having the habit of seeking health information, 36 (65.5%; p-value = 0.003), and longer time on ART, 47 (59.5%; p-value = 0.017), are related to a higher risk for illiteracy (Table 5).

**Table 5.** Distribution of literacy level of people living with HIV according to clinical and behavioral variables – Recife, PE, Brazil, 2019.

**Factor assessed**	**Literacy**		PR*	(95%) CI**	**p-value**
	**Illiterate**	**Literate**			
**Controlled medication use**					
Yes	27(52.9%)	24(47.1%)	1.14	0.85–1.53	0.396^1^
No	117(46.4%)	135(53.6%)	1.00	–	
Interruption of ART*					
Yes	50(54.9%)	41(45.1%)	1.25	0.98–1.59	0.081^1^
No	92(44.0%)	117(56.0%)	1.00	–	
**Search for information**					
Yes	108(43.5%)	140(56.5%)	1.00	–	**0.003** ^1^
No	36(65.5%)	19(34.5%)	1.50	1.18–1.91	
**ART use time**					
6 months to 5 years	48(39.0%)	75(61.0%)	1.00	–	**0.017** ^1^
6 to 10 years	47(59.5%)	32(40.5%)	1.52	1.15–2.03	
> 10 years	49(48.5%)	52(51.5%)	1.24	0.92–1.68	

^1^p-value of Pearson’s chi-square test for independence. *Antiretroviral therapy; **Prevalence ratio; ***Correlation Index.

## DISCUSSION

The term HL was used for the first time, referring to a concern with the increase in the complexity of health services that were not accompanied by people’s knowledge about their use^(15)^. Almost half a century later, the same seems to be happening with regard to HIV, with the increase in costs for its control not accompanied by population education measures. Although HL alone does not determine better therapeutic compliance or greater self-care, it appears to influence better health outcomes^(3)^.

When it comes to gender, women are more affected by the low literacy and social impacts that this deficit provides. Being a woman, having less schooling and income are associated with lower HL and greater impact of the social gradient, resulting in less autonomy and possibilities of choices about one’s own body and sexual freedom^(16)^. A study carried out in the south of Brazil highlights a high death rate in young women living with HIV, demonstrating marked social inequalities, given that these women were also responsible for supporting minor children, living in unsanitary housing, in addition to living in poverty situation^(17)^.

The invisibility of women among the priority groups in the fight against HIV is reproduced in the heterosexual men cluster. These findings should be highlighted, since it is among women that the greatest risk for inadequate HL was found. Two points of discussion deserve to be highlighted: emphasis on HIV prevention among priority groups, which may be relegating other groups to a secondary position in the preventive paradigm; a lower perception of risk among heterosexual men and women, resulting in lower prevention in these groups^(18)^. The sum of these factors is worrying, as it is reproduced in a scenario of low literacy among women.

Regarding the level of formal education and income, there is an association demonstrated in the literature that the higher the education, the better the employment and income opportunities, i.e., the better the social position or the better the social gradient. In other words, the more favorable the socioeconomic situation, the better the chance of having good health^(19)^. Although HL does not have a direct association with education level, better health outcomes are associated with better education levels, which can be confirmed by the findings described here.

Considering the social gradient, which interferes with living with HIV and, similarly, the levels of HL in a feedback interaction between them, social macrodeterminants linked to socioeconomic conditions stand out, such as material deprivation, few years of formal education, low income and underprivileged social class. They interfere with the results presented by PLHIV, suggesting that the social gradient persists in continuous care, in which those people with greater livelihood deprivation are less able to maintain compliance with ART and an undetectable viral load^(19,20)^. Remission of viral load is essential to end HIV infections, however it needs general measures to reduce social disparities.

The existing relationships between living with HIV and the social determinants of health (SDH) are demonstrated in the literature and verified in this study. In turn, HL is influenced by such determinants, but also influences them in a complex and multifactorial relationship. Although this interaction does not occur exclusively, HL can be a direct determinant of health, mediator of such relationships and moderator between the other determinants and health. Having a vast HL and, to a lesser extent, adequate functional literacy is considered a determinant in direct health, as well as an important inducer in the promotion of healthy habits and access to better opportunities to solve gaps and health problems^(21)^.

Regarding compliance with ART throughout HIV treatment, the association between greater HL and strict compliance with drug therapy was found in this study, in the same way that the findings of a study carried out in the city of São Paulo showed that the lower the HL, the greater the difficulties in complying with treatment^(22)^. Health professionals have an essential role in health education and in the promotion of measures that support compliance, given that the central focus of public policy to reduce new cases of HIV infection is viral load remission, only being possible through compliance with ART.

In the same direction, there are international efforts, in which the objective of ending HIV as a public health problem by 2030 proposes that social inequalities be fought and intersectoral actions are expanded. Achieving the 95-95-95 target should be the target of all countries in the world, and it will only be possible with the availability of testing and treatment in a timely manner, in addition to viral load remission. However, obtaining results depends on risk perception and timely testing^(23)^. As well as compliance with drug therapy, it is also necessary for PLHIV to assume an active self-care posture through the adoption of a healthy way of life, attendance at scheduled appointments, examinations and consultations, when directed, performance of complementary therapies, harm reduction practices and use of support networks to cope with their health condition. The center of care is PLHIV, so an active attitude in the treatment itself should be encouraged by health professionals.

Supported self-care is one of the tools that guide nursing care, starting from the problems presented by the user and the establishment of goals through the nurse-patient interaction during consultations and educational nursing interventions^(2,24)^. With regard to educational measures in health, nurses play a prominent role, as they are the professional present in every health unit where nursing care is provided, in addition to developing activities at all levels of complexity of the health system. Nurses educate in health, teaching users to develop self-care, a necessary premise for the control of chronic diseases such as HIV.

The result of this interaction should be the critical HL, i.e., the HL that allows the subject’s autonomy to make decisions about their health, and the center of care is PLHIV. Although the data found in this study showed a similar distribution of self-care between people with adequate and insufficient literacy, the increase in the self-care score showed an improvement in compliance with ART. Furthermore, most of the PLHIV interviewed who presented adequate HL also had strict compliance with ART. Such findings lead to the conclusion that better health outcomes depend on subjects’ autonomy. Social and educational barriers interfere with the development of HL and, consequently, reduce self-care; therefore, they must be recognized by the nurse during the nursing consultation, being redeemed together with patients^(25,26)^.

The central role of nurses in assisting PLHIV is highlighted with the Ministry of Health’s proposal to reorganize the care model through the decentralization of care from the SCS to Primary Health Care (PHC). Bearing in mind that PHC is a health space that is closest to people’s lives, guaranteeing the therapeutic bond, it provides comprehensive and longitudinal care, transcending the moment of HIV diagnosis. Holistic follow-up of cases through home visits allows nurses to intervene, to adopt preventive measures and develop health promotion activities^(27)^. All these measures are essential for the development of critical HL.

Also noteworthy among the findings is the positive impact that the search for health information brings, when related to HL, as opposed to treatment time. It was evidenced that, the longer the bonding time to continuous care in SCS, the less adequate HL. Unreliable sources of information, added to possible cognitive deficits caused by the HIV virus, can lead people to incorporate questionable recommendations, especially for those with low HL^(28)^. Encouraging HL in health has a positive impact on continuity of care, compliance with ART and reduction of missed appointments^(29)^. Self-care supported by nurses, with remission of doubts and construction of a care plan built together with the client, added to interventions throughout the treatment, is a priority for continuous quality care.

Receiving continuous care for years, as well as demonstrating inadequate health HL, is worrying, since contact with health professionals should promote greater literacy over time, a fact not evidenced in this study. The use of personal skills related to HL is mediated by the environment where its use is necessary. In this case, it is necessary to apply educational interventions that give people the opportunity to demonstrate acquired skills and capacities of individuals. In addition to this, the use of simple language and the reduction of the complexity of health systems represent ways to reduce the impacts of low literacy^(2)^.

Limitations are related to the availability of patients linked to SCS, not being possible to investigate cases of abandonment of ART, which could elucidate better conditions of low literacy and the impacts of these conditions on treatment. Another limitation was the comparison with data from other national studies, which reinforces the need to develop research that leads to an understanding of the reality of the different regions of Brazil.

## CONCLUSION

We found that the highest self-care scores were associated with strict compliance with the treatment of HIV infections. In the same sense, people with an excellent level of self-care and who showed strict compliance with medication had adequate literacy.

We emphasize that HL, as a conceptual basis for health promotion, is an effective health care strategy for PLHIV. In this sense, its adoption as a public policy can contribute to empowerment, self-care and expansion of access to health services.

Therefore, the importance of the quality of continuous care for PLHIV is highlighted, as well as the emphasis on the role of nurses’ educator in the construction of critical literacy that promotes autonomy, especially for patients on ART who have been treated for a longer time in SCS. Although interventions that seek to improve HL cannot by themselves lift people out of unfavorable conditions, they can reduce the impacts between social determination and health inequities when it comes to living with HIV.
